# Analysis of Laboratory Repeat Critical Values at a Large Tertiary Teaching Hospital in China

**DOI:** 10.1371/journal.pone.0059518

**Published:** 2013-03-14

**Authors:** Dagan Yang, Yunxian Zhou, Chunwei Yang

**Affiliations:** 1 Department of Laboratory Medicine, The First Affiliated Hospital, College of Medicine, Zhejiang University, Hangzhou, China; 2 School of Nursing, Zhejiang Chinese Medical University, Hangzhou, China; University of Houston, United States of America

## Abstract

**Context:**

As a patient safety measure, laboratories are required to have a critical values policy by regulatory agencies. Unfortunately, little information is available on repeat critical values for the same analyte(s) on the same patient.

**Objective:**

To investigate the occurrence and distribution of repeat critical values and the relationship between the frequency of such values and patient outcome to provide information for hospitals on improving reporting policies.

**Methods:**

Eleven laboratory critical value lists, including chemistry and hematology analytes, were selected from a tertiary hospital in China in the year 2010. The distribution and interval time for each repeat critical value were calculated. Serum potassium and platelet count were used as examples to illustrate the relationship between the frequency of the repeat critical values and patient outcome.

**Results:**

All test items on the critical value list were prone to the occurrence of repeat critical values. On average, each patient who experienced critical values had 2.10 occurrences. The median interval time for each repeat critical value varied, with most being longer than 8 hours. For those patients who had repeat critical values of serum potassium and platelet count, along with the increased frequency, the patients had a longer hospital stay and a generally worse outcome.

**Conclusions:**

Patient can have a number of repeat critical values and the frequency of these values is closely related to patient outcome. A careful evaluation is warranted if a laboratory chooses to adopt a policy of not reporting each repeat critical value.

## Introduction

A laboratory critical value refers to an extremely abnormal laboratory test result which may be life threatening if treatment is not initiated immediately. This concept was first introduced by Lundberg in 1972 and has been widely adopted worldwide [1]. The Joint Commission International, ISO 15189 and the College of American Pathologists all have clear requirements on the identification, handling, documentation and auditing of laboratory critical value [2]–[4]. The Chinese Hospital Association also made the reporting of critical value one of the National Patient Safety Goals from 2007 to 2011 [5]. It is expected that all laboratories establish a critical value list with ranges based on hospital size and specialties. Also important to consider are the impact on laboratory and physician time as well as user input on which values are included [1]–[5]. The number of critical values will influence laboratory workload, clinical care and treatment. Excluding the time nurses and physicians require when dealing with the critical value, laboratory personnel need 4–6 minutes to complete a critical value call for hospital inpatients and 11–14 minutes to complete a call for outpatients [6], [7].

There are some common practices for laboratory critical value policy, such as the list, the range and the reporting procedure [8]. However, little information is available on repeat critical values or subsequent critical values for the same analyte(s) on the same patient. Studies by Howanitz et al. [9], [10] implied that both whole blood sodium and total serum calcium alerts can occur in the same patient more than once. However, it was not clear which laboratory tests were prone to repeat critical values and there were very few data on the distribution of such values. Additionally, we have no idea whether patients who had more repeat critical values on a certain test item had a worse outcome compared to those who only had one critical value. This would have some implication on the reporting of subsequent repeat critical values. The literature is full of inconsistencies on this issue and different hospitals have their differing practices, such as calling for only the first critical value, calling for each critical value, calling for worsening values, or calling for critical values once per interval of time [6], [7], [11]–[14]. Reporting of laboratory repeat critical values is an issue worthy attention because of important regulatory, legal and clinical concerns.

We analyzed 11 laboratory critical value test items, including chemistry and hematology analytes, from a large tertiary hospital in China in the year 2010. Our aim was to explore the incidence and distribution of repeat critical values, including single analyte repeat critical values, multiple analytes repeat critical values and the interval time for each single analyte repeat critical values. We also used serum potassium and platelet count as examples to retrospectively analyze the relationship between the frequency of repeat critical values and the length of hospital stay and patient outcome. All of this information will serve to guide laboratories on how to improve reporting policies for repeat critical values.

## Materials and Methods

### Setting

The First Affiliated Hospital, School of Medicine, Zhejiang University, is an adult comprehensive tertiary teaching hospital in Hangzhou, Zhejiang Province, P.R. China. There are currently 2,800 beds in the hospital. Its clinical laboratory is a national clinical key specialty lab, with sub-specialties such as clinical hematology, biochemistry, and immunology. The laboratory attained ISO 15189 certification in October 2011. The critical value list including the test items and ranges from 2010 is shown in Table 1. This list was typical of tertiary hospitals in China. It was based on reports from the College of American Pathologists [6], [15] and the patient safety requirements of the Chinese Hospital Association [5], with consultation from relevant clinical experts and was approved by the hospital Medical Quality Committee.

**Table 1:** The critical value list and its ranges.

		Critical Value Range	
Analyte	Unit	Low Threshold	High Threshold
pH	-	7.15	7.58
pCO_2_	mm Hg	20	75
pO_2_	mm Hg	40	-
Glucose	mmol/L	2.5	27.8
Potassium	mmol/L	2.80	6.50
Sodium	mmol/L	115	160
Calcium (Total)	mmol/L	1.6	3.5
Prothrombin time	s	-	30 (non-severe liver disease ward) or 50 (severe liver disease ward)
Partial thromboplastin time	s	-	80
WBC count	10^9^/L	1.5	50.0
Platelet count	10^9^/L	20 (non- hematological ward) or 10 (hematological ward)	1000

### Critical Value Reporting

In 2010, the process of critical value reporting was as follows: when a laboratory technician detects a critical value, he/she will validate the critical result by repeat testing or by checking the quality of the specimen and any historical results for the patient. For inpatients, the result will then be posted on the patient's Electronic Medical Record. The Laboratory Information System and the Electronic Medical Record will send a short message and a screen reminder to the physician. The Medical Order System will also send out a screen reminder to the ward nurses. The laboratory technician then phoned the nurses on the patient's floor to notify them of the patient's critical value. The nurses will then remind the physician to address the critical value result. For outpatients, the laboratory technician will inform either the patient or his/her family member using the contact phone number required for all outpatients during registration. If not successful, the physician who ordered the test will be informed. If this fails, the emergency center of the hospital will be informed of the situation. In addition, there is a reminder on the laboratory test report indicating that this is a critical result and that it is recommended that the patient contact his or her physician immediately. All phone contacts, including time, contact person and contact details will be recorded. According to the policy, we assess the critical value scope, incidence, timeliness of reporting, and effectiveness each year.

### Ethics Statement

This study was approved by the ethics committee of the First Affiliated Hospital, College of Medicine, Zhejiang University, China, and was in accordance with the Helsinki declaration. The institutional review board waived the need for written informed consent from participants.

### Source of Data

Using Powerbuilder 11.5 software, the raw data test results from January 1, 2010 to December 31, 2010 for 11 critical value analytes were obtained from the Laboratory Information System and saved as a Visual FoxPro DBF database. The contents of the data included specimen number, unique patient identification number, patient name, department, diagnosis, analyte, result, and report time.

### Data Processing and Analysis

String data (data containing explanatory information such as “twice result”, “recheck”) were removed from the test results. Only numeric data were left so that test results could be compared and computed using symbols such as “>” and/or “<”. In addition, false positive critical values, such as those caused by specimen hemolysis, were removed. The raw data were calculated and summarized using Standard Query Language, and the data were then processed using an EXCEL spreadsheet to show the distribution and P_10_, P_50_, and P_90_ interval times for each repeat critical value. Serum potassium and platelet count were chosen as examples. Data on diagnosis, length of hospital stay and patient outcome were extracted from the Electronic Medical Record to analyze the relationship between the frequency of the repeat critical values and patient outcome.

### Statistical Analysis

Statistical analyses were performed using SPSS version 15.0 for Windows. Frequencies, medians, quartile and percentages were used for descriptive statistics. The variable for patient outcome was categorized using four levels: healed, improved, not improved, and died. This was coded as 1, 2, 3 and 4 before statistical analysis. The relationships between the frequency of repeat critical values and the length of hospital stay and patient outcome were evaluated by Kruskal-Wallis nonparametric tests. Man-Whitney U tests were used as post hoc tests to compare between two groups. Because there were three groups for comparison, *p* = 0.05/3 = 0.0167 was used as the critical level of significance.

## Results

### The Incidence and Distribution of Critical Values

In the year 2010, there were approximately 3,170,000 tests for the 11 test items included in the critical value list with the total number of patients being approximately 259,000. If a patient visited both the outpatient department and inpatient department of the hospital it was calculated as two cases. Of these, 10,516 patients had 30,400 person time critical values, an incidence rate of 0.96%. Among these patients, 6,912 cases and 24,754 person time critical values were from the inpatient department and 3,604 cases and 5,646 person times critical values were from the outpatient department.

### The Distribution of Single Analyte Repeat Critical Values

The distribution of single analyte repeat critical values is shown in Table 2. It is evident that all the 11 test items were prone to repeat critical values. The main tests for repeat critical values were white blood cell (WBC) and platelet count. Blood glucose was the least likely test to have repeat critical values. On average, each patient who experienced a critical value had it occur 2.10 times.

**Table 2:** The distribution of single analyte repeat critical values.

Analyte	Frequency						Total patient^a^	Total person times	Average time	Repeat critical value (%)
	1	2	3	4	5	>5				
pH	347	89	43	18	9	31	537	1057	1.97	35.4
pCO_2_	479	147	66	46	26	89	853	2501	2.93	43.9
pO_2_	968	157	67	25	18	42	1277	2177	1.70	24.2
Glucose	399	37	12	3	1	4	456	566	1.24	12.5
Potassium	1356	320	120	61	27	29	1913	2947	1.54	29.1
Sodium	145	56	22	11	15	15	264	583	2.21	45.1
Calcium	402	94	37	21	7	24	585	1055	1.80	31.3
Prothrombin time	1396	438	136	48	31	21	2070	3203	1.55	32.6
Partial Thromboplastin time	1677	266	101	49	41	85	2219	3657	1.65	24.4
WBC count	948	419	250	158	106	360	2241	7159	3.19	57.7
Platelet count	1046	382	203	120	70	208	2029	5495	2.71	48.5
Total	9163	2405	1057	560	351	908	14444	30400	2.10	36.6

a For practical reasons, each critical result here was calculated as one patient. For instance, if a patient had repeat critical values for three analytes, it was calculated as three patients. Therefore, the total number of patients (14444) in the table was more than the real number of patients (10516).

### The Distribution of Multiple Analytes Repeat Critical Values

There were 523 cases of repeat critical values for two analytes in the same patient. The combinations were mainly WBC and platelet count (372 cases), prothrombin time and partial thromboplastin time (33 cases), and platelet count and partial thromboplastin time (12 cases). There were 61 cases of repeat critical values for three analytes in the same patient; the combinations were mainly serum potassium and WBC and platelet count (11 cases), partial thromboplastin time and prothrombin time and platelet count (11 cases), and pH and pCO_2_ and pO_2_ (8 cases). Additionally, there were also several cases of repeat critical values for four or more analytes in the same patient (data not shown).

### The Interval Time for Each Repeat Critical Value

The P_10_, P_50_ (median), and P_90_ interval times for each repeat critical value are shown in Table 3. The results indicated that the median intervals between different analytes varied greatly, with pH having the shortest interval (4 hours) and prothrombin time having the longest interval (120 hours).

**Table 3:** The Interval for Each Repeat Critical Value (Hours).

Analyte	P_10_	P_50_	P_90_
pH	1	4	149
pCO_2_	1	7	96
pO_2_	2	24	214
Glucose	2	24	216
Potassium	3	17	260
Sodium	4	16	52
Calcium	5	22	250
Prothrombin time	5	120	2226
Partial thromboplastin time	2	44	193
WBC count	17	68	312
Platelet count	9	49	240

### Clinical data analysis of patients with repeat critical values of serum potassium

**Figure 1:**
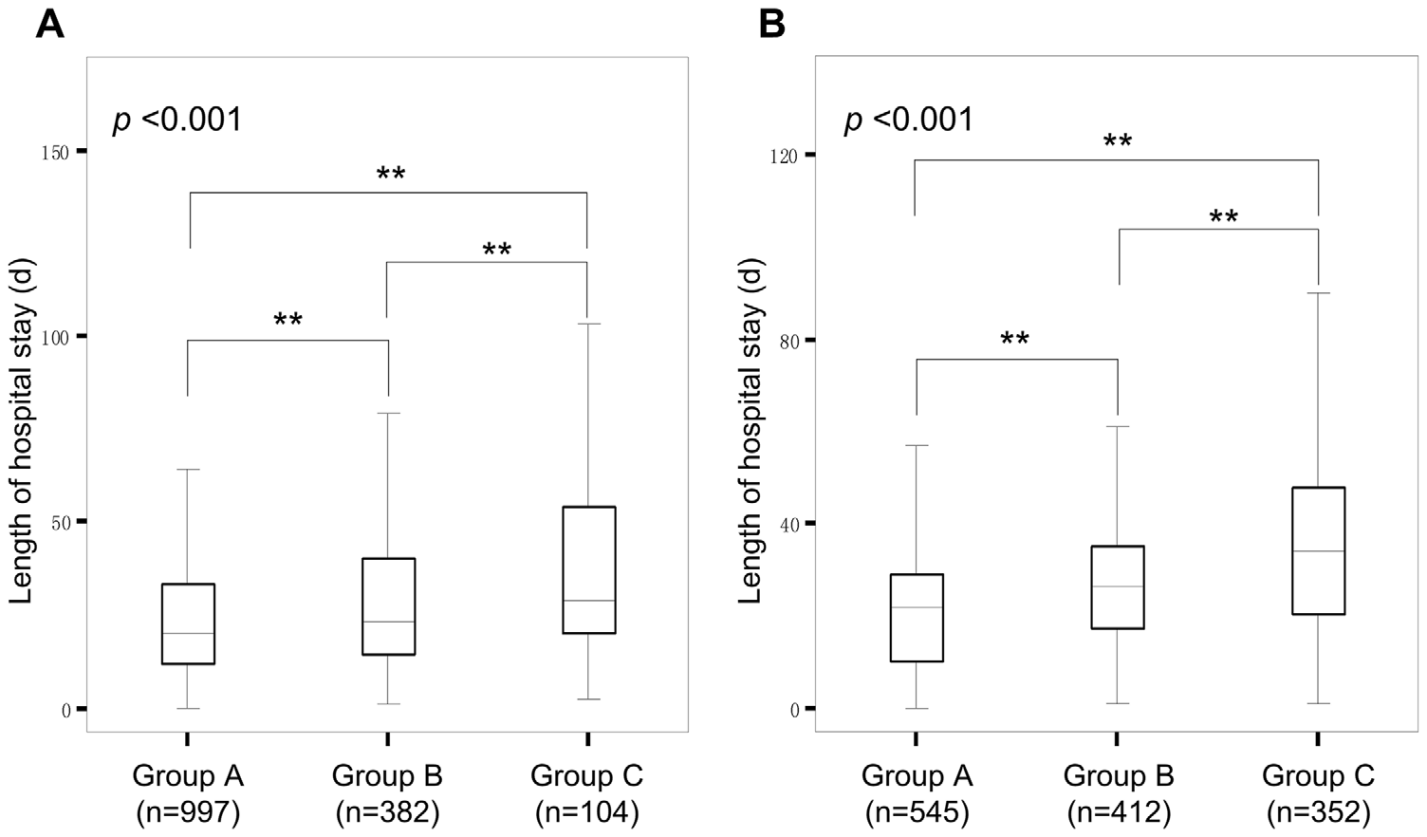
The relationship between the frequency of critical values and the length of hospital stay. Note: (A) critical values of serum potassium. (B) critical values of platelet count. The patients were divided into 3 groups based on the frequency of the critical values: group A (1 time), group B (2–3 times), group C (≥4 times). Statistics: box plots showing the median, quartiles, and range. Kruskal-Wallis nonparametric test, *p*-values shown. Man-Whitney U tests (** p<0.0033).

**Figure 2:**
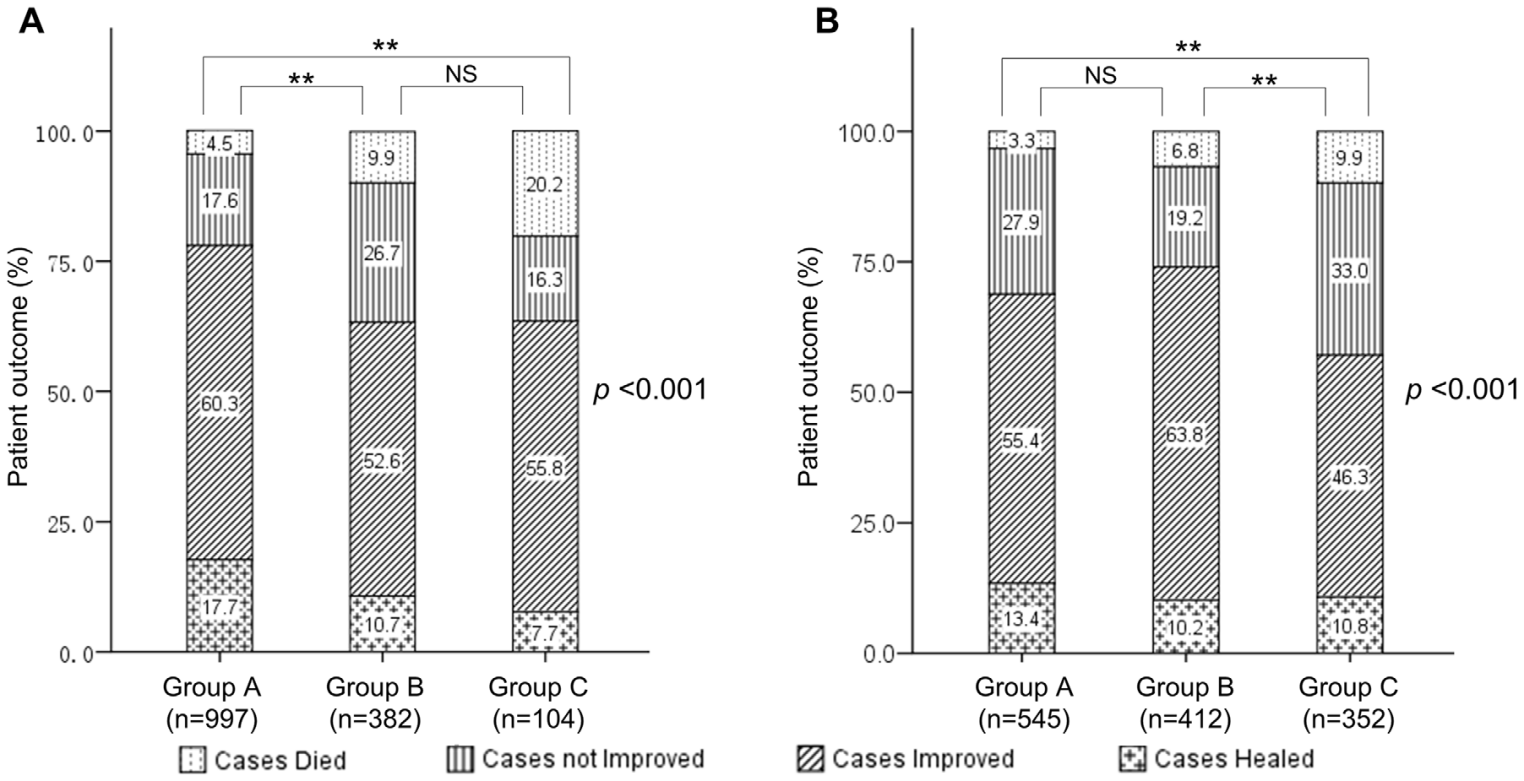
The relationship between the frequency of critical values and patient outcomes. Note: (A) critical values of serum potassium. (B) critical values of platelet count. The patients were divided into 3 groups based on the frequency of the critical values: group A (1 time), group B (2–3 times), group C (≥4 times). Statistics: Kruskal-Wallis nonparametric test, *p*-values shown. Man-Whitney U tests (**, *p*<0.0033. NS, not significant).

There were 557 cases of repeat critical values for serum potassium in the laboratory in 2010. Of these, 71 cases were from the outpatient clinic (42 from the emergency center and 13 from the renal center) and 486 cases were from the inpatient department (130 from the hepatitis ward, 58 from the hepatopancreatobiliary surgical ward, 54 from renal ward, 50 from intensive care unit, 37 from endocrine ward, and 34 from hematologic ward). The main diagnoses for inpatients with repeat critical values for serum potassium were kidney diseases, cancer, hepatitis B with liver cirrhosis, leukemia, hypokalemia, and infection. Patients were divided into 3 groups: group A for those with only one critical value for serum potassium, group B for those with two or three critical value for serum potassium and group C for those with no less than four critical value for serum potassium. Kruskal-Wallis tests showed that there were significant differences between groups in both the length of hospital stay and patient outcome (*p*<0.001) (Figure 1A, Figure 2A). Post hoc comparisons using a Mann-Whitney U test revealed that groups B and C had significantly longer hospital stays and a worse patient outcomes than group A (all *p*<0.0033). Group C had significant longer hospital stays than group B, but did not differ significantly in patient outcome from group B (*p*>0.0167). Along with the increased frequency of critical value for serum potassium, the mortality rate increased from 4.5% to 20.2%.

### Clinical data analysis of patients with repeat critical values of platelet count

There were 983 cases of repeat critical values for platelet count in the laboratory in 2010. Of these, 219 cases were from the outpatient clinic (104 from the emergency center and 80 from the hematology clinic) and 764 cases were from the inpatient department (360 from the hematology ward, 97 from the hepatitis ward, 92 from the intensive care unit, and 75 from the hepatopancreatobiliary surgical ward). The main diagnoses for those inpatients with repeat critical values for platelet count were platelet diseases, acute and chronic leukemia, liver cirrhosis, gastrointestinal bleeding, and cancer. Patients were divided into 3 groups: group A for those with only one critical value for platelet count, group B for those with two or three critical value for platelet count and group C for those with no less than four critical value for platelet count. Kruskal-Wallis tests showed that there were significant difference between groups in both the length of hospital stay and patient outcome (*p*<0.001) (Figure 1B, Figure 2B). Post hoc comparisons using Mann-Whitney U test revealed that group B and group C had significantly longer hospital stays than group A. Group C had significant longer hospital stays than group B (all *p*<0.0033) and also had significantly worse patient outcomes than groups A and B. Group A did not differ significantly in patient outcome from group B (*p*>0.0167). Along with the increased frequency of critical value for platelet count, the mortality rate increased from 3.3% to 9.9%.

## Discussion

In 2010, the incidence rate of critical value in the clinical laboratory of our hospital was 0.96%, slightly higher than what was reported in the literature but still within the limit of less than 1% [6], [15]. The high rate of critical value might relate to the economic level of China. Without insurance coverage, some patients from the countryside may not seek medical treatment until their condition is severe. Additionally, as a tertiary hospital, many patients are transferred here from local hospitals, so we have a relatively high number and percentage of severe and emergency patients and thus more critical values.

As Table 2 shows, all analytes in the critical value list had repeat critical values and on average each patient with a critical value had it occur 2.10 times. In the literature, it was reported that, on average, a patient with critical value for serum sodium could have it occur 3.53 times, and this number was 3.31 for serum calcium [9], [10]. Our findings suggest that the occurrence of repeat critical values were related to certain diseases and wards. For instance, our patients with repeat critical values for platelet count were mainly from the hematological ward. This suggests that laboratories should make every effort to consult with clinicians in these wards to tailor the handling of repeat critical values in the patient population and to meet the needs of both clinicians and patients. As another measure, a population specific critical value range could be considered. For instance, in 2008, the lower threshold of platelet count in our hospital was changed to 10×10^9^/L for patients in the hematological ward after approval from the Hospital Quality Control Committee [8]. Without this change, the number of critical values for platelet count in this study would have been even more. We also have population specific critical value ranges for prothrombin time in the severe liver disease ward. Until now, we did not have different standards for WBC thresholds aimed at patients from the hematological ward. As a result, the average time of critical value for WBC was 3.19, with an approximate repeat critical value rate of 57.7%. This put WBC on the top of the list for both the number of critical value and repeat critical values. Although it is good practice to set population specific critical value ranges, it is difficult to do so in some wards, such as the intensive care unit and emergency center because of the wide range of diseases treated therein.

Blood glucose had the lowest occurrence of repeat critical values among our tests. From Table 2, we can see that, on average, the critical value of glucose occurred 1.24 times. This was likely related to the adoption of point of care tests for blood glucose in our hospital. A glucometer was widely used for initial screening and later for monitoring of blood glucose by registered nurses, due to the advantages of simplicity and speed. Point of care test results were not included in the scope of critical value management in our hospital. This caused few repeat critical values of blood glucose in the results.

We also found many multiple analytes repeat critical values in the same patient during our analysis. The main combinations of two analyte repeat critical values were WBC and platelet count and partial thromboplastin time and prothrombin time. These reflected problems of the same system, such as the hematological system or the blood coagulation system. These tests were usually performed by the same laboratory division and the same laboratory technician can complete the reporting of multiple analyte critical results, thus reducing the influence on the workload of both the clinic and laboratory. When a patient has three or more analytes repeat critical values, such as the combination of potassium and WBC and platelet count or partial thromboplastin time and prothrombin time and platelet count, it usually indicates an extreme disorder of multiple systems, an even more critical situation for the patient. When there are more than two analytes repeat critical values for the same patient, it usually involves two or more laboratory divisions and technicians. In this case, several phone calls are required to complete the critical results reporting. Given the severe situation, more clinical attention should be paid to patients with multiple analytes repeat critical values. Currently there are no guidelines on how to report such critical results; we suggest that each critical value should be reported to promote patient safety.

The interval between the critical values of the same analyte and same patient is a very important part of the decision of whether to report all repeat critical values or not. If the intervals are short, the medical staff are still on the same shift, and they are clear about the patient's condition, it may not be necessary to report all the repeat critical values. On the contrary, if the intervals are long, it is usually necessary to report the critical values in order to remind physicians of the patient's changed condition. Our results showed that the median interval of repeat critical values for different analytes varied from 4 to 120 hours, with most analytes having a median interval longer than 8 hours. In this case, different laboratory technicians, nurses and physicians would be involved, and some of them may not know the state of previous critical values. We suggest these repeat critical values should be reported each time to give clinicians adequate information. Although we chose 8 hours as the reporting cut-off point, others may feel more comfortable with a longer time frame, such as the first of a series of values within 12 or 24 hours requiring notification. It is good practice to establish an individualized reporting strategy aimed at each specific analyte based on its median interval. For instance, blood pH (4 hours) and pCO_2_ (7 hours) had the shortest median interval of repeat critical values. We consider it fine not to call repeat critical values of these two analytes within 8 hours.

There is little data on the relationship between the frequency of repeat critical values and patient outcome. This information is important when optimizing reporting policy. Two indicators were chosen based on the literature: length of hospital stay and patient outcome expressed as healed, improved, not improved or died [9], [10]. In 2010, we reported all repeat critical values with a combination of sending short messages to physicians and making phone call to the ward nurses regardless of the previous results. Using serum potassium and platelet count as examples, our results demonstrated that along with increased frequencies of repeat critical values, patients had a longer hospital stay and an increased mortality rate.

Potassium plays an important role in maintaining cellular polarization and is critical for the transmission of electrical impulse through the myocardium. Hypokalemia is associated with heightened ventricular excitability and an increased risk of ventricular arrhythmias. Hyperkalemia disturbs cardiac conduction and can cause arrhythmias and precipitate cardiac arrest [16]. Goyal et al. reported that serum potassium values of less than 3.5 or more than 5.0 mEq/L were associated with a high adverse cardiac event and mortality rate in patients with acute myocardial infarction [17]. Hypokalemia was also found to be a predictor of serious peri- and postoperative arrhythmias [18]. As shown in Figures 1A and 2 A, an increase in the number of repeat critical value s for serum potassium was closely related to an increase in mortality (from 4.5% to 9.9% and 10.2%) with a decrease in healing rating (from 17.7% to 10.7% and 7.7%). Similarly, platelets help the blood clot. If the number of platelets is too high, blood clots can form which may obstruct blood vessels and result in events such as stroke, myocardial infarction, or pulmonary embolism. However, if the number of platelets is too low excessive bleeding can occur. Studies have found a strong negative correlation between the admission platelet count and mortality for ICU patients [19]. Our results also indicate the critical condition of those patients with repeat critical values of platelet count. This is in accordance with the original definition of critical value by Lundberg, where critical condition implies that constant clinical attention is required.

From a laboratory management perspective, more attention should be paid to the management of repeat critical values. In the literature, it is quite common that not all repeat critical values are reported. This is concerning, as there is very little research evidence showing that it is safe to do so. Clinicians may not be fully clear on why certain critical values are not reported, which can lead to undesirable consequences if intervention is delayed. A repeat low platelet count can be a critical value even if it is an expected finding because it can influence patient management and may prompt physicians to withhold or delay necessary invasive interventions, reduce the intensity of anticoagulation, or order prophylactic platelet transfusion. A Q-track study reported that the practice of mandatory notification to caregivers of repeat inpatient critical values was associated with improved overall performance in critical value reporting when compared to institutions that had a policy to not call back repeat critical values [20]. In addition, reporting all repeat critical values is a good indication of an increased level of vigilance, which is essential for sustaining patient safety efforts [20]. Based on our results, at least regarding serum potassium and platelet count, the practice of not calling each repeat critical value is unwarranted and thus requires careful evaluation.

We believe our findings are representative of critical patients in large tertiary hospitals in developing countries. Due to the unique setting, they may not be representative of small or rural hospitals in the US or other Western countries. To the best of our knowledge, this is the first report on the distribution and interval of repeat critical values from China, and one of very few such reports internationally. We also know of no previous studies reporting the association of the frequency of repeat critical values on patient outcome. Although this study shed some light on reporting policies for repeat critical values, it remains uncertain which policy is most beneficial for patient outcome. Future studies testing the effectiveness of different policy approaches are highly recommended.

### Conclusion

We retrospectively analyzed the laboratory repeat critical values at a large tertiary hospital in China. Patients can have a number of repeat critical values for different tests, and the frequencies of such values are closely related to patient outcomes. It is necessary for each laboratory to have a policy on how to handle repeat critical values based on a number of factors such as interval time and the nature of the test item, combined with reporting methods and hospital characteristics. This will clarify laboratory technician responsibilities and establish consistency in performance. Theoretically, it is possible not to call each repeat critical value, and it is often difficult to develop a policy that is tailored to different situations, especially when a laboratory technician has limited information available and a short period of time to make a reporting decision. A generic policy that covers all patients is practical because it is easiest for technicians to implement and causes no errors in judgment. To ensure patient safety, a careful evaluation is warranted if a laboratory adopts a policy of not to call each repeat critical value.
